# Transvaginal diverticulectomy for urethral diverticulum in a young female: case report

**DOI:** 10.11604/pamj.2025.50.6.46029

**Published:** 2025-01-06

**Authors:** Moussaab Rachid, Ghassane El Omri, Omar Lazrak, Younes Houry, Abdeljalil Heddat

**Affiliations:** 1 Department of Urology, Cheikh Khalifa International University Hospital, Mohammed VI University of Health Sciences (UM6SS), Casablanca, Morocco

**Keywords:** Diverticulum, urethral diseases, diverticulectomy, case report

## Abstract

This case highlights the unique presentation of a 36-year-old woman with a urethral diverticulum (UD), a rare and under-recognized condition in women, diagnosed following a three-year history of lower urinary tract symptoms (LUTS) unresponsive to standard treatment and complicated by dyspareunia. Clinical examination revealed a tender anterior vaginal wall mass, and the diagnosis was confirmed by retrograde urethrocystography and magnetic resonance imaging (MRI), which revealed no additional complications. The patient underwent transvaginal diverticulectomy via an inverted U-shaped incision, with meticulous multi-layered closure of the periurethral fascia and vaginal wall. Postoperative recovery was uneventful, and the patient experienced complete resolution of symptoms. This case contributes to the scientific literature by underscoring the diagnostic challenge of UD due to its nonspecific symptoms and rarity, which often lead to delayed diagnosis and mismanagement. It emphasizes the importance of maintaining a high index of suspicion in women presenting with recurrent or persistent LUTS and dyspareunia. The successful surgical outcome further highlights the efficacy of transvaginal diverticulectomy as the gold-standard treatment, provided a meticulous technique is employed to minimize complications. The main takeaway from this case is the critical role of early recognition, thorough clinical evaluation, and imaging in diagnosing UD to ensure timely and effective management, ultimately improving patient quality of life.

## Introduction

A urethral diverticulum, or suburethral pouch, is defined as a herniation of the urethral mucosa through the smooth muscle fibers comprising the urethral wall. It forms a cul-de-sac that often communicates with the urethral lumen via a collar and protrudes into the vaginal wall [1,2]. First described by Lee in 1984 [3], it is a rare condition with a poorly understood pathophysiology. Its diagnosis is primarily clinical, confirmed by radiological imaging, and its management is surgical. We present the case of a 36-year-old female patient with a urethral diverticulum who underwent surgical diverticulectomy.

## Patient and observation

**Patient information:** a 36-year-old married mother of two (G2P2) presented to the outpatient department with irritative lower urinary tract symptoms (LUTS) that had been ongoing for three years. She had no significant medical or surgical history.

**Clinical findings:** on clinical examination, the patient was hemodynamically and respiratorily stable and remained afebrile. Examination of the abdomen and perineum revealed no abnormalities. A vaginal examination identified a renal mass on the anterior vaginal wall, which was tender but not painful on palpation. The remainder of the urogenital examination was unremarkable.

**Timeline:** symptoms began three months prior to admission with the gradual onset of irritative lower urinary tract symptoms, including pollakiuria and urgency. The patient had three outpatient consultations, during which she was treated with oral antibiotics for presumed uncomplicated cystitis. However, her urinary symptoms not only worsened but were also accompanied by the development of dyspareunia, prompting her to seek specialist advice.

**Figure 1:**
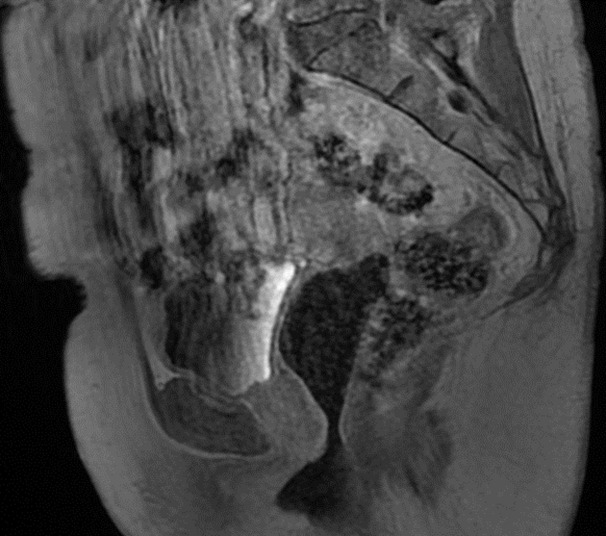
sagittal section of a pelvic magnetic resonance imaging without complications related to the urethral diverticulum

**Diagnostic assessment:** given the mass identified during clinical examination, retrograde urethrocystography was conducted, revealing an additive image in the middle part of the urethra, suggestive of a urethral diverticulum. A pelvic MRI was subsequently performed, revealing no abnormalities or complications related to the urethral diverticulum (Figure 1). From a biological standpoint, no abnormalities were detected in the complete blood count, C-reactive protein levels, renal function tests, hemostasis parameters, or urine cytobacteriological analysis.

**Figure 2:**
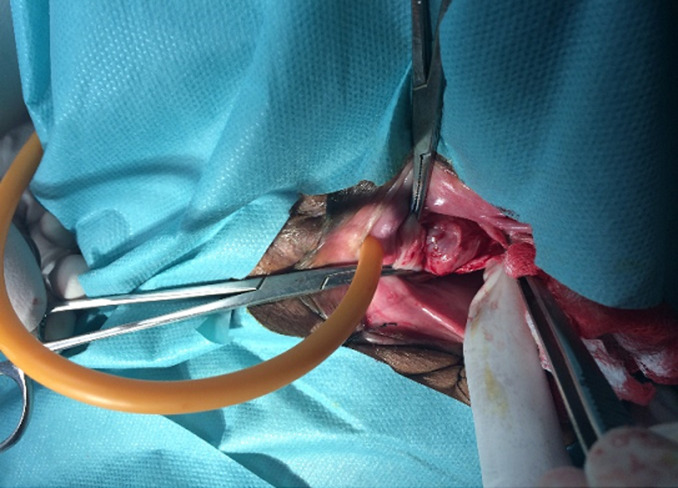
intraoperative image of a urethral diverticulum after dissection

**Therapeutic interventions:** the patient underwent surgery one week after the consultation. The procedure involved the removal of the diverticulum through a transvaginal diverticulectomy. It was performed under spinal anesthesia with the patient in the gynecological position, following antibiotic prophylaxis. After the placement of a bladder catheter, the diverticulum was accessed via an inverted U-shaped vaginal incision. The procedure proceeded with the dissection of the diverticular neck, which was then closed and secured, followed by closure of the periurethral fascia and the anterior vaginal wall (Figure 2).

**Follow-up and outcome of interventions:** the patient had an uneventful post-operative recovery. The bladder catheter was removed on the fifth day, and the patient was discharged seven days after the operation. The patient had a follow-up consultation one month after the operation, during which it was observed that the incision was clean, and there were no signs of parietal infection. A positive clinical progress was noted, and LUTS disappeared.

**Patient perspective:** “at first, I thought I was suffering from recurrent cystitis for which I had taken several different antibiotics. But with no improvement and the onset of dyspareunia, my symptoms began to seriously affect my quality of life. But once the diagnosis had been made and the surgical treatment carried out properly, I felt reassured. I'd like to thank the urology and radiology teams who took such good care of me, both before and after the operation”.

**Informed consent:** an informed consent for the publication of this article in a journal was obtained from the patient.

## Discussion

Urethral diverticulum (UD) is a rare condition in women, with a reported prevalence ranging from 0.6% to 6% in the general population [4]. It typically presents between the third and fifth decades of life, as seen in our case of a 36-year-old patient [5]. The pathophysiology of UD remains poorly understood, with both congenital and acquired mechanisms proposed. Congenital forms are thought to result from remnants of embryological structures, such as Gartner´s ducts [6], while acquired cases are associated with repeated infections, inflammation, and obstruction of periurethral glands, leading to cyst formation and subsequent rupture into the urethra. Risk factors include multiparity, obstetric trauma, and prior urethral or pelvic surgery [3]. The clinical presentation of UD is highly variable, often leading to diagnostic delays. While the classic triad of symptoms-dysuria, dyspareunia, and post-micturition dribbling-is described in the literature, it is seen in only 5%-20% of cases [2]. Instead, many patients present with non-specific lower urinary tract symptoms (LUTS), recurrent urinary tract infections (UTIs), or a palpable anterior vaginal wall mass [3]. Our patient experienced irritative LUTS and dyspareunia, symptoms that significantly impacted her quality of life. This underscores the importance of maintaining a high index of suspicion, especially in women with persistent or recurrent LUTS unresponsive to standard treatment.

Imaging plays a pivotal role in confirming the diagnosis and guiding management. While retrograde urethrocystography has been historically used, magnetic resonance imaging (MRI) is now considered the gold standard due to its superior soft tissue contrast and ability to delineate the anatomy and complexity of the diverticulum [7]. In our case, the MRI revealed no additional complications, and the imaging findings supported the diagnosis of UD. Surgical excision via transvaginal diverticulectomy remains the definitive treatment, offering symptom resolution in 83%-97% of cases [8]. The surgical approach involves careful dissection and multi-layered closure to prevent complications such as fistulas, strictures, or recurrence [8]. Our patient underwent an inverted U-shaped incision with meticulous closure of the periurethral fascia and vaginal wall, achieving a favorable outcome.

Postoperative complications, including stress urinary incontinence (SUI), recurrent UTIs, or urethrovaginal fistulas, have been reported in 3%-20% of cases [9]. However, no such complications were observed in our patient, highlighting the importance of meticulous surgical technique and perioperative care. Although rare, malignancy within a UD should be considered, particularly in cases with atypical symptoms such as hematuria or significant pain. Histopathological examination of excised specimens is essential to rule out underlying malignancy [10]. This case underscores the necessity of early recognition and accurate diagnosis of UD to prevent prolonged patient suffering and ensure timely intervention. It also emphasizes the importance of a multidisciplinary approach involving urologists and radiologists to achieve optimal outcomes. Further research is needed to refine diagnostic strategies and assess long-term outcomes following surgical management.

## Conclusion

Diverticulum of the female urethra is a rare and under-recognized condition with etiopathogenesis that remains poorly understood. Functional symptoms are primarily characterized by micturition disorders, recurrent urinary tract infections, and dyspareunia. Diagnosis, which is essentially clinical, is confirmed by retrograde and voiding cystourethrography (UCRM) or, in some cases, transvaginal ultrasound. Surgical excision via transvaginal diverticulectomy in the dorsal position remains the gold standard for treatment.
